# Catalytic Intermediate Crystal Structures of Cysteine Desulfurase from the Archaeon *Thermococcus onnurineus* NA1

**DOI:** 10.1155/2017/5395293

**Published:** 2017-04-24

**Authors:** Thien-Hoang Ho, Kim-Hung Huynh, Diem Quynh Nguyen, Hyunjae Park, Kyoungho Jung, Bookyo Sur, Yeh-Jin Ahn, Sun-Shin Cha, Lin-Woo Kang

**Affiliations:** ^1^Department of Biological Sciences, Konkuk University, Hwayang-dong, Gwangjin-gu, Seoul 05029, Republic of Korea; ^2^Department of Life Science, Sangmyung University, 7 Hongji-dong, Jongno-gu, Seoul 03016, Republic of Korea; ^3^Department of Chemistry and Nano Science, Ewha Womans University, 52 Ewhayeodae-gil, Seodaemun-gu, Seoul 03760, Republic of Korea

## Abstract

*Thermococcus onnurineus* NA1 is an anaerobic archaeon usually found in a deep-sea hydrothermal vent area, which can use elemental sulfur (S^0^) as a terminal electron acceptor for energy. Sulfur, essential to many biomolecules such as sulfur-containing amino acids and cofactors including iron-sulfur cluster, is usually mobilized from cysteine by the pyridoxal 5′-phosphate- (PLP-) dependent enzyme of cysteine desulfurase (CDS). We determined the crystal structures of CDS from *Thermococcus onnurineus* NA1 (ToCDS), which include native internal aldimine (NAT), gem-diamine (GD) with alanine, internal aldimine structure with existing alanine (IAA), and internal aldimine with persulfide-bound Cys356 (PSF) structures. The catalytic intermediate structures showed the dihedral angle rotation of Schiff-base linkage relative to the PLP pyridine ring. The ToCDS structures were compared with bacterial CDS structures, which will help us to understand the role and catalytic mechanism of ToCDS in the archaeon *Thermococcus onnurineus* NA1.

## 1. Introduction

**Scheme 1:**
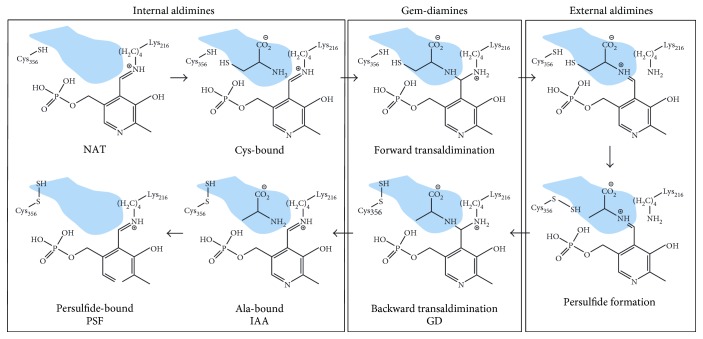
Simplified catalytic mechanism of CDS with the conserved Cys and Lys residues and PLP cofactor in the active site. For the catalytic residues, the corresponding residue numbers of ToCDS are labelled. The substrate channel is represented as a blue shade. Four catalytic intermediate structures of ToCDS (NAT, GD, IAA, and PSF) determined in this study are labelled in bold black letters.

*Thermococcus onnurineus* NA1 is a sulfur-reducing hyperthermophilic organism living in a strictly anaerobic condition of a deep-sea hydrothermal vent area at high temperatures between 80 and 100°C [1]. In the sulfur-rich extreme environment, *Thermococcus onnurineus* NA1 can use elemental sulfur (S^0^) as a terminal electron acceptor for energy via dissimilatory sulfur metabolism and produce H_2_S as a by-product. In the absence of sulfur, *Thermococcus onnurineus* NA1 can also use CO, formate, or soluble starch and produce a different by-product of biohydrogen (H_2_) [2]. Sulfur is also essential for diverse biomolecules such as thiol-containing cofactors, iron-sulfur ([Fe-S]) clusters, molybdopterin, and tRNA thionucleosides [3–5], which are synthesized via assimilatory sulfur metabolism. Cysteine desulfurase (CDS; EC 2.8.1.7) is an important enzyme for the assimilatory sulfur metabolism, where it performs the following desulfuration reaction [3, 6, 7] (Scheme 1):

(1) $$\begin{array}{c} L‐Cysteine+\left[enzyme\right]‐cysteine\;↔\;L‐alanine+\left[enzyme\right]‐S‐sulfanylcysteine \end{array}$$

The catalytic mechanism of bacterial CDSs consists of two steps [8]. In the first step, the sulfur atom of cysteine is transferred into the side chain of catalytic cysteine residue of CDS, which forms a persulfide-bound intermediate (Cys-S-SH). In the second step, the sulfur atom is transferred to diverse acceptor proteins like [Fe-S] cluster scaffold proteins and sulfurtransferases.

The [Fe-S] clusters, one of the earliest catalysts in the biomolecule evolution, work as versatile electron carriers in redox reactions, regulatory sensors, stabilizers of protein structure, and chemical catalysts [9, 10]. In bacteria, three multiprotein systems which are nitrogen fixation (NIF), iron-sulfur cluster (ISC), and mobilization of sulfur (SUF) are involved in the [Fe-S] cluster assembly, in which various CDSs are essential to transfer sulfur from free cysteine to diverse sulfur intermediate acceptors such as TusA, IscU, SufE, SufU, and CsdE [5, 11–13].

*Thermococcus onnurineus* NA1 exists near the root of the evolutionary tree of life and has a limited number of 1976 predicted genes [1], which is almost half the genes in *Escherichia coli*. *Thermococcus onnurineus* NA1 has a CDS gene (*ToCDS*), and no sulfur intermediate acceptor gene has been identified. Many archaeons found near solfataric hydrothermal vents do not have a CDS gene [14]. Currently, little is known about archaeal assimilatory sulfur metabolism for biomolecule synthesis. The differences in CDS genes have been of interest with respect to the evolution of sulfur cycle in life [15].

CDS belongs to the pyridoxal 5′-phosphate- (PLP-) dependent enzyme family. PLP-dependent enzymes exist ubiquitously: 1.1%, 1.3%, and 0.5% of genes in archaeon, bacteria, and eukaryote, respectively, are PLP-dependent enzymes [16]. PLP provides the core catalytic power via the PLP pyridine ring working as an electron sink [17]. The internal aldimine Schiff-base linkage between PLP and the amino group of active site Lys residue switches back and forth to the external aldimine Schiff-base linkage between PLP and the substrate amino group in the middle of catalysis. The PLP-driven catalytic power enables PLP-dependent enzymes to perform more than 140 different enzyme activities of oxidoreductases, transferases, hydrolases, lyases, and isomerases, which include five out of six general classes of all enzymes [16].

In the catalysis of PLP-dependent enzymes, the transaldimination reaction is strictly conserved and happens at least two times in forward and reverse directions: the forward reaction is from the internal aldimine to the external aldimine and the reverse reaction is vice versa (Scheme 1). Recently, the conformational change of PLP, especially the dihedral angle between the PLP pyridine ring and the Schiff-base linkage, was proposed to play an essential role in the transaldimination reaction in a bacterial L-serine hydratase (XometC) [18]. In this study, we cloned the *ToCDS* gene from *Thermococcus onnurineus* NA1 and determined the crystal structures of ToCDS alone and in complex with catalytic intermediate ligands from four different crystals, which were then compared with *Escherichia coli* CDS structures.

## 2. Materials and Methods

### 2.1. Cloning

The open reading frame sequence encoding ToCDS protein from *Thermococcus onnurineus* NA1 was amplified by PCR using the genomic DNA isolated from *Thermococcus onnurineus* NA1 as a template according to a previously described method [19]. The sequences of the oligonucleotide primers were designed based on the data on genome sequences of *Thermococcus onnurineus* NA1 from the NCBI website. Forward (5′-CCC CC**G CTA GC**A TGA TTC CGG AGG ATG TTA-3′) and reverse (5′-CCC CC**G CGG CCG C**TT AAG TCT TCA GAC CTT TTA-3′) primers were designed to introduce *Nhe*I and *Not*I restriction sites (bold), respectively. The PCR-amplified DNA fragments were purified using a PCR purification kit (Bioneer, Republic of Korea), inserted into the same restriction enzyme digested pET29b-His-Tev (pET29bHT) vector which was modified from the original pET-29b vector (Novagen, Germany) to have seven histidine residues and a TEV cleavage site at the N-terminus of gene product in order to facilitate protein purification. The expression vector pET29bHT-*ToCDS* was transformed into *Escherichia coli* BL21(DE3) and plated on Luria Bertani (LB) [20] agar containing 50 *μ*g ml^−1^ kanamycin. A kanamycin-resistant colony was selected, and plasmid DNA from the transformant was isolated using a plasmid purification kit (Favorgen, Taiwan). DNA sequencing to confirm the cloning was carried out at the Macrogen facility (Seoul, Republic of Korea).

### 2.2. Overexpression and Purification

*Escherichia coli* BL21(DE3) cells containing pET29bHT-*ToCDS* were grown at 310 K to optical density at 600 nm (OD_600_) of 0.6 in LB medium supplemented with 50 *μ*g ml^−1^ kanamycin. The protein expression of ToCDS was induced by the addition of isopropyl *β*-D-1-thiogalactopyrasnoside (IPTG) to a final concentration of 0.5 mM. The cells were cultured at the same temperature, 310 K. After overnight growth, cells were harvested by centrifuging at 6000 ×g (Vision VS24-SMTi V5006A rotor, Republic of Korea) for 20 min at 277 K. The resultant cell pellets were resuspended in ice-cold lysis buffer (25 mM Tris-HCl pH 7.5, 300 mM NaCl, 15 mM imidazole, 20% (*v*/*v*) glycerol, and 3 mM *β*-mercaptoethanol) and disrupted using a sonicator (Sonomasher, Republic of Korea). The crude cell extract was centrifuged for 30 min at 21,000 ×g (Vision VS24-SMTi V508A rotor, Republic of Korea) at 277 K to remove cell debris. The supernatant containing soluble ToCDS was applied onto Ni-NTA resin (Novagen, Germany) previously equilibrated with the lysis buffer, and affinity purification was performed according to manufacturer's protocol. All protein purification steps were carried out at 277 K. The column was washed with buffer B consisting of 25 mM Tris-HCl pH 7.5, 1 M NaCl, 15 mM imidazole, and 20% (*v*/*v*) glycerol. The elution buffer containing 250 mM imidazole was used to elute the 7 × His-tagged ToCDS. The eluted ToCDS was dialyzed for 8 h at 277 K against the dialysis buffer (25 mM Tris-HCl pH 7.5, 15 mM NaCl, 3 mM *β*-mercaptoethanol, and 20% (*v/v*) glycerol). Further purification was carried out in a HiTrap Q anion-exchange column (GE Healthcare, USA) equilibrated in buffer A (25 mM Tris-HCl pH 7.5, 15 mM NaCl, 3 mM *β*-mercaptoethanol, and 20% (*v*/*v*) glycerol). ToCDS was washed and eluted with a gradient of 0 to 100% buffer B. The purification protocol for the recombinant form of ToCDS consistently yielded more than 50 mg of ToCDS per liter of culture medium, and the protein exhibited a single band on SDS-PAGE at approximately 44 kDa. For crystallization, the protein solution was concentrated using centrifugal filters (Amicon® Ultra-15, MWCO 10 kDa, Germany) to a final concentration of 13 mg ml^−1^ in a buffer consisting of 25 mM Tris-HCl pH 7.5 and 15 mM NaCl.

### 2.3. Crystallization and X-Ray Data Collection

Initial crystallization screening was carried out at 287 K by the sitting-drop vapor-diffusion method in 96-well Intelli-plates (Art Robbins, USA) using a Hydra II e-drop automated pipetting system (Matrix, USA) and screening kits from Hampton Research (Crystal Screen Cryo, Crystal Screen Lite, Crystal Screen HT, Index HT, and SaltRx HT), MD1-46 (Morpheus™, USA), and Wizard Precipitant Synergy (Emerald Bio, USA). Two kinds of ToCDS (13 mg ml^−1^ ToCDS alone and 12 mg ml^−1^ ToCDS containing 5 mM L-cysteine and 1 mM PLP) were set up for crystallization. The protein solution (0.5 *μ*l) was mixed with 0.5 *μ*l of reservoir solution and equilibrated against 50 *μ*l of reservoir solution at 287 K.

In two days, crystals of ToCDS protein alone (crystal I) were observed in condition A11 (crystal I condition) of MD1-46 (Morpheus, USA) containing 0.03 M MgCl_2_, 0.03 M CaCl_2_, 15% (*v*/*v*) glycerol, 15% (*w*/*v*) PEG 4000, and 0.1 M Tris (base)/Bicine pH 8.5. The fully grown crystals were flash-cooled at 100 K in liquid nitrogen with the cryoprotection solution of the crystal I condition with additional 5% glycerol. For the trials to obtain reaction-intermediate structures, crystal I was transferred into the solution of crystal I condition supplemented with 5 mM L-cysteine and 1 mM PLP for various durations. The catalytic intermediate structure of gem-diamine (GD) was determined from the crystal soaked for 1 h (crystal II). The same cryoprotection solution of crystal I was used to flash-cool crystal II at 100 K in liquid nitrogen.

Similarly, in two days, the crystals of ToCDS (crystal III) were observed in condition C2 (crystal III condition) of MD1-46 (Morpheus, USA) containing 0.03 M NaNO_3_, 0.03 M Na_2_HPO_4_, 0.03 M (NH_4_)_2_SO_4_, 15% (*v*/*v*) ethylene glycol, 15% (*w*/*v*) PEG 8000, and 0.1 M imidazole/MES pH 6.5 with the ToCDS protein sample containing 5 mM L-cysteine and 1 mM PLP. The fully grown crystals were flash-cooled at 100 K in liquid nitrogen with the cryoprotection solution of the crystal III condition with additional 5% glycerol.

The crystals of ToCDS (crystal IV) were observed in the additional crystallization solution (crystal IV condition) containing 10% (*v*/*v*) isopropanol, 5% (*w*/*v*) PEG 8000, and imidazol/HCl pH 5.9 from the ToCDS protein sample containing 5 mM L-cysteine and 1 mM PLP. The fully grown crystals were flash-cooled at 100 K in liquid nitrogen with the cryoprotection solution of the crystal IV condition with additional 20% glycerol. X-ray data were collected at the beamline 7A and 5C at the Pohang Light Source (PLS), Republic of Korea. Diffraction data were integrated and scaled using Denzo and Scalepack, respectively [21].

### 2.4. Structure Determination

The autoindexing program [21] showed that the crystal of ToCDS alone (crystal I), which represents a native (NAT) structure, belonged to the space group P2_1_2_1_2_1_ with unit-cell parameters *a* = 67.0, *b* = 92.5, and *c* = 145.4 Å. The three screw axes of P2_1_2_1_2_1_ were confirmed with systematic absences. Diffraction data were collected to 2.6 Å resolution. The structure of ToCDS alone was solved using the molecular replacement method. The *MOLREP* [22] from the *CCP4* program package [23], using the CDS structure from *Synechocystis* sp. PCC6803 (PDB entry 1T3I [24]; 42.8% sequence identity) as a search model, was successful and showed a dimer in the asymmetric unit. The initial *R* factor from the molecular replacement search was 54.5%. After the molecular structure modeling and refinement by Coot [25] and *REFMAC5* [26], the *R* factor decreased to 19.3% and the free *R* factor was 25.9%. The determined native ToCDS structure was used as a template to solve the ligand-bound complex structures. Structure-based multiple sequence alignment was performed in *T-Coffee* [27] and then presented using the *ESPript* server [28]. Molecular graphics were created using PyMOL [29].

## 3. Results

### 3.1. Primary Structure of ToCDS

**Figure 1:**
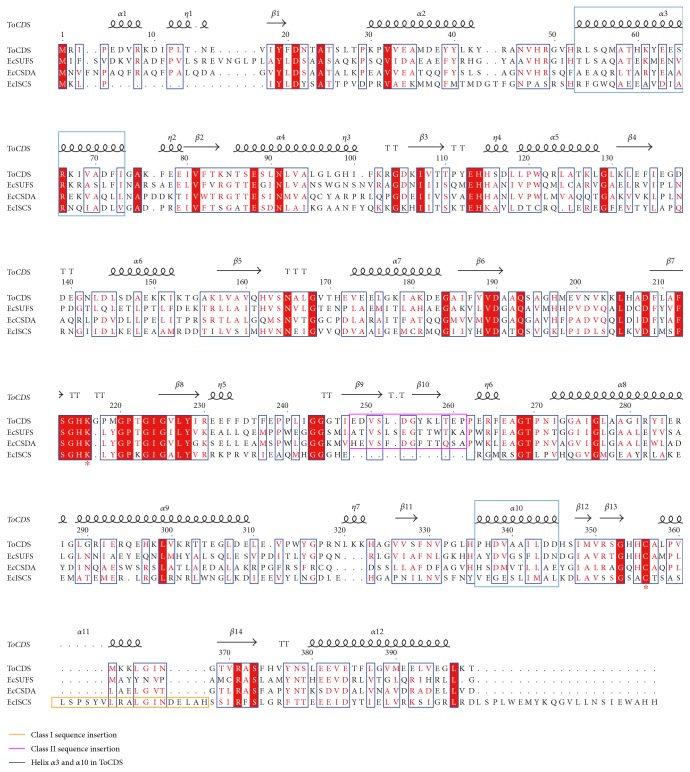
Structural sequence alignment of ToCDS with EcCDSs. ToCDS is CDS from *Thermococcus onnurineus* NA1; EcISCS, EcSUFS, and EcCSDA are IscS, SufS, and CsdA from *E. coli*. Conserved catalytic Cys and Lys residues are labelled with an asterisk. The class I sequence insertion is shown in an orange rectangle and the class II sequence insertion in a pink rectangle. The helix *α*3 and *α*10 of ToCDS and the corresponding helices of EcCDSs are shown in blue rectangles.

All known CDSs show similarities in the amino acid sequence and the three-dimensional structure. However, local structural differences with characteristic reactivities have been used to assign CDSs into two classes [3, 30]: class I includes IscS-like sequences and class II includes SufS- and CsdA-like sequences (Figure 1). Class I CDSs contain a sequence insertion of more than 10 residues just after the conserved catalytic Cys, and class II CDSs contain a shorter insertion after the conserved catalytic Lys. The primary sequence of ToCDS fits into class II; furthermore, ToCDS has the sequence identity of 39% with EcSufS, 33% with EcCsdA, and 28% with EcIscS in 399 residues.

### 3.2. Overall Structure of Native ToCDS

**Figure 2:**
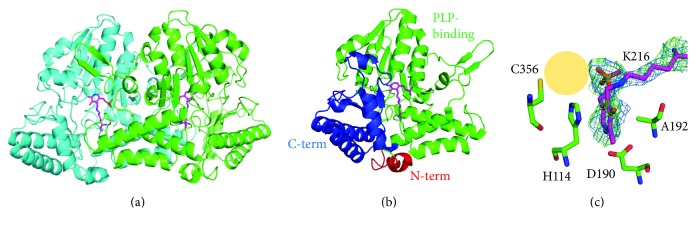
Structure of native ToCDS (NAT) in crystal I. (a) Overall internal aldimine structure of ToCDS dimer. (b) Protomer structure of ToCDS. (c) Active site structure of ToCDS. Substrate cysteine-binding site is shown as an orange shade. Omit maps of internal aldimine of PLP and Lys216 are shown. The omit maps show the 2Fo-Fc electron density map (contoured at 1.0 *σ*; blue) and Fo-Fc electron density map (contoured at 3.0 *σ*; green).

The crystal structure of native ToCDS (NAT) was determined to be 2.6 Å (Table 1). There was a dimer with a twofold symmetry in the asymmetric unit (Figure 2(a)). A protomer of ToCDS comprised three domains (Figure 2(b)). The N-term domain contained residues 1–16 and included two parallel *α*-helices. The larger central domain having PLP binding site contained residues 17–285 and comprised nine *α*-helices surrounding a nine *β*-stranded, mainly parallel *β*-sheet. The C-term domain (residues 286–399) contained four *α*-helices, together with a two-stranded antiparallel *β*-strands.

ToCDS formed an obligate functional dimer, with 3882 Å^2^ buried at the dimer interface out of 16,979 Å^2^ of each protomer's solvent accessible surface. The two active sites were located in the center of the PLP-binding domain at the dimer interface. PLP was Schiff-base linked with Lys216 at the active site. The PLP pyridine ring was well stacked with His114 and bound to Ala192 with van der Waals interaction at the opposite side (Figure 2(c)). 5′-phosphate of PLP was tightly bound via hydrogen bonds with the nearby residues of Ser87, His215, Ser213, and Thr268.

### 3.3. Structure Comparison between ToCDS and EcCDSs

**Figure 3:**
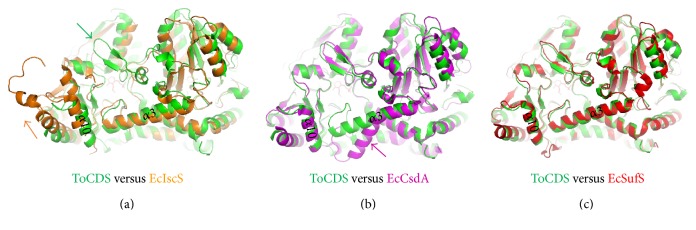
Structure comparison between ToCDS and EcCDS structures. (a) Superimposed structures of ToCDS (green) and EcCDS IscS (orange). The *β*-turn structure of ToCDS is labelled by a green arrow. The up-shifting C-terminal domain of IscS is shown by an orange arrow. (b) Superimposed structures of ToCDS (green) and EcCDS CsdA (purple). The bent helix *α*3 of CsdA is labelled by a purple arrow. (c) Superimposed structures of ToCDS (green) and EcCDS SufS (red).

**Figure 4:**
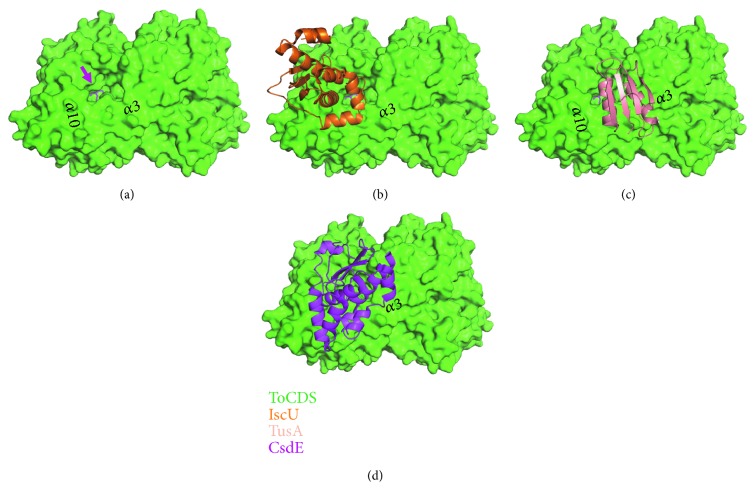
Proposed acceptor protein-binding site of ToCDS. (a) Surface representation of ToCDS. The substrate channel is labelled by a pink arrow and PLP is shown in pink. Helix *α*3 and *α*10 are labelled. (b) IscS/IscU complex structure is superimposed on ToCDS structure and only IscU is represented as a cartoon. (c) IscS/TusA complex structure is superimposed on ToCDS structure and only TusA is represented as a cartoon. (d) CsdA/CsdE complex structure is superimposed on ToCDS structure and only CsdE is represented as a cartoon.

*Escherichia coli* has three CDS proteins: EcIscS (class I), EcCsdA (class II), and EcSufS (class II), of which crystal structures were determined alone and in complex with [Fe-S] scaffolds or sulfur acceptor proteins like IscU, TusA, and CsdE [8, 31]. ToCDS structure was superimposed and compared with the EcCDS structures in complex with their sulfur acceptor proteins (Figures 3 and 4). Overall core folds of ToCDS and three EcCDSs were conserved but showed differences in regions. In the EcCDS complex structures, the acceptor proteins were bound close to the mouth of substrate channel via interaction with the helix *α*3 or *α*10 (the number of secondary structure follows the ToCDS numbering) (Figures 1 and 4(a)). In EcIscS structure, there was no *β*-turn structure of ToCDS (Ile247-Thr258 in ToCDS as class II sequence insertion) forming the upper mouth of the substrate channel. Instead, a loop (Ala327-Leu333 in EcIscS) existed at the opposite side of the substrate channel, and the C-terminal domain was relatively shifted up (Figure 3(a)). IscU was bound via interactions with the helix *α*10 (Figure 4(b)). TusA was bound via interactions with the helix *α*3 (Figure 4(c)). In EcCsdA structure, the long *α*3 helix was bent outwards and generated a big hole into the PLP active site (Figure 3(b)). CsdE was bound between the bent helix *α*3 and the helix *α*10 of the C-terminal domain via the main interactions with the helix *α*10 (Figure 4(d)). EcSufS showed the most similar structure with ToCDS, including the proposed acceptor-binding site and had an extra *β*-turn loop at the N-terminal domain (Figure 3(c)). No acceptor protein-bound complex structure of EcSufS is yet determined.

### 3.4. Catalytic Intermediate ToCDS Structures

**Figure 5:**
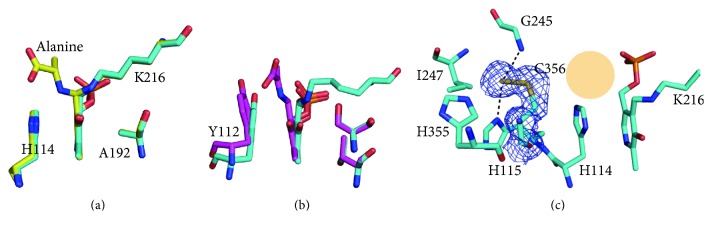
Comparison of ToCDS and XometC catalytic intermediate structures. (a) Superimposed ToCDS NAT (cyan) and GD (yellow) structures with nearby residues. (b) Superimposed XometC NAT (cyan) and external aldimine with alanine (purple) structures with nearby residues. (c) Active site structure of ToCDS PSF. The 2Fo-Fc electron density map is contoured at 1.0 *σ* (blue).

We studied if the catalytic mechanism of ToCDS was conserved with that of the bacterial CDSs. To determine catalytic intermediate structures of ToCDS with substrate, product, or reaction intermediate ligands, we soaked and cocrystallized substrate L-cysteine with native ToCDS crystals and determined three different complex structures of ToCDS (see Figure S1 in Supplementary Material available online at https://doi.org/10.1155/2017/5395293). The complex ToCDS structures included the gem-diamine structure with persulfide-bound Cys356 (Figure S1b), the internal aldimine structure with existing alanine (IAA) with persulfide-bound Cys356 (Figure S1c), and the internal aldimine structure with persulfide-bound Cys356 (PSF) (Figure S1a and Figure 5(c)) as follows the catalytic order of reactions (Scheme 1()).

### 3.5. Gem-Diamine (GD) Structure

In crystals II and III, three protomers of GD structure were determined, which showed the clear electron densities of PLP-bound alanine, PLP, and Schiff-base linkage bonds (Figure S1b). The persulfide group was removed from substrate cysteine and transferred to Cys356. When we superimposed the ToCDS GD structure into the ToCDS NAT structure, the conformations of the PLP pyridine ring and His114 of both structures were well conserved (Figure 5(a)). In the catalytic intermediate structures of XometC [18], a PLP-dependent enzyme of L-serine hydratase, both the PLP pyridine ring and Tyr112 (His114 in ToCDS) showed the tilting conformational changes (Figure 5(b)). However, no tilting of the PLP pyridine ring and His114 was observed in ToCDS.

**Figure 6:**
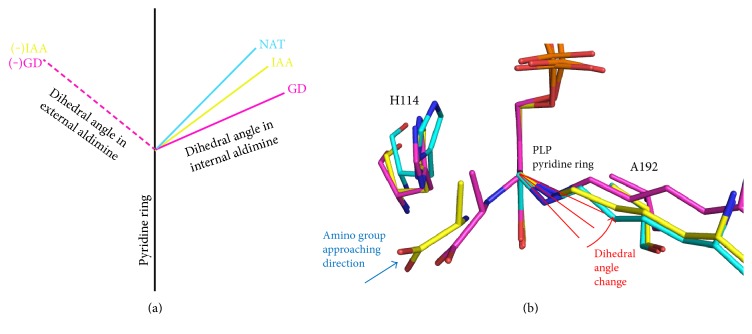
Dihedral angles of ToCDS protomers in crystals I, II, III, and IV. (a) Averaged dihedral angle of each catalytic state. Positive dihedral angles are for the internal aldimine. Negative dihedral angles are for the external aldimine. (b) Superimposed structures of ToCDS NAT (cyan), GD (purple), and IAA (yellow). Structures were superimposed based on the position of the PLP pyridine ring.

The dihedral angle between the PLP pyridine ring and Schiff-base linkage bond with Lys216 (internal dihedral angle) in the ToCDS GD structure got wider than that in the ToCDS NAT structure by almost 20° (Figure 6 and Table 2). The dihedral angle between the PLP pyridine ring and the Schiff-base linkage with alanine (external dihedral angle) was found to be narrower than the inner dihedral angle (Figure S2a and Table 2). Two amino groups of Lys216 and product alanine had the similar distances of approximate 2.9 Å with the hydroxyl group in an equilateral triangle form. The hydroxyl group of PLP was proposed as catalytic base to deprotonate the incoming substrate amino group in XometC [18]. In *Escherichia coli*, NAT (PDB ID: 1jf9) and GD (in complex with selenocysteine; PDB ID: 1kmk) structures of CsdB (EcNifS_CsdB), which is a NifS-like CDS [32], were determined [33]. Similar to ToCDS intermediate structures, the internal dihedral angle of the EcNifS_CsdB GD structure was wider than that of the EcNifS_CsdB NAT structure by 35°. The amino group of substrate selenocysteine in EcNifS_CsdB GD structure also existed close to the hydroxyl group of the PLP pyridine ring.

### 3.6. Internal Aldimine Structure with Existing Alanine (IAA)

In crystal III, the internal aldimine structure with existing alanine (IAA) was determined in protomer A, which showed the clear electron densities of alanine near PLP but not connected with PLP (Figure S1c). The persulfide group was transferred and bound to Cys356, which showed the same conformation with that in the GD structure. The internal dihedral angle between the PLP pyridine ring and Schiff-base linkage bond was in the middle between those of the NAT and GD structures. The proposed imaginary external dihedral angle, between the PLP pyridine ring and the amino group of alanine in the IAA structure, was almost the same as the external dihedral angle in the GD structure. However, the amino group of product alanine in the IAA structure was further away from PLP by 0.7 Å, when compared to that of the GD structure. Two amino groups, in Lys216 and product alanine, and the hydroxyl group of PLP made an almost perfect triangle (Figure S2b).

### 3.7. Persulfide-Bound Cys356 (PSF) Structure

In crystal IV, the internal aldimine structure with persulfide-bound Cys356 was determined at 1.9 Å (Figure S1a and Figure 5(c)). In protomer A, persulfide-bound Cys356 was shown and positioned in the same location as the GD and IAA structures. In protomer B, Cys356 was not shown due to flexible conformation. PSF structure is the structure after the sulfur transfer from cysteine to ToCDS and release of alanine product from ToCDS. The persulfide on Cys356 was bound with His115 and Gly245 via hydrogen bonds and His355 and Ile247 via van der Waals interactions (Figure 5(c)). The bound persulfide faced the mouth of the substrate channel, which is the putative acceptor protein-binding site. The persulfide position proposes the rotational conformational change of the Cys356 side chain after picking up the sulfur atom from substrate cysteine in the active site. The persulfide-bound EcNifS_CsdB structure (PDB ID: 1kmj) [33] also showed the same rotated persulfide-bound side chain of active site Cys residue like ToCDS GD, IAA, and PSF structures, which implies that the interactions between the terminal persulfide and nearby active site residues are important for the conformational change. The conformation of PLP and Schiff-base linkage of the ToCDS PSF structure was well conserved with that of the ToCDS NAT structure (Figure 5(a)).

### 3.8. Structure Comparison of Catalytic Intermediate Structures of ToCDS

We compared the catalytic intermediate structures and the dihedral angles between the PLP pyridine ring and the internal and external amino groups of Lys216 and alanine of ToCDS (Figure 6 and Table 2). Overall conformations of the intermediate structures were well conserved. However, the internal dihedral angle showed flexible conformational changes from 38° to 69°: the internal dihedral angle got wider in the order of NAT, IAA, and GD structures. The external dihedral angle and proposed external dihedral angle in GD and IAA structures stayed almost similar as −51°.

## 4. Discussion

We determined crystallographic snapshots of catalytic intermediate structures of ToCDS (Scheme 1). Transaldimine reaction is the conserved critical step of all PLP-dependent enzymes. Recently, the dihedral angle change between the PLP pyridine ring and Schiff-base linkage was proposed to play an essential role in the transaldimination reaction of XometC based on the catalytic intermediate crystal structures and high-level computational calculations [18] (Figure S3). The conformational change of the dihedral angle had two main roles. First, it attracted the nucleophilic attack of substrate amino group on the Schiff-base linkage of PLP. Second, the hydroxyl group of the PLP pyridine ring had the catalytic base and acid role in the deprotonation of the incoming substrate amino group and the concerted proton transfer between two amino groups in the GD structure.

The catalytic intermediate structures of ToCDS showed similar dihedral angle changes with XometC during the catalytic steps. The PLP-dependent catalytic mechanism was well conserved. The internal dihedral angle of ToCDS changed from 45° in the NAT structure to 66° in the GD structure on average. In our understanding, the steric hindrance from the incoming substrate amino group of cysteine pushed away the Schiff-base linkage amino group of Lys216 without tilting the PLP pyridine ring due to the tight binding. In the GD and IAA structures, the amino group of ligand was positioned just next to the hydroxyl group of the PLP pyridine ring, which is a suitable conformation to deprotonate the incoming substrate amino group and perform the proton transfer between two amino groups of substrate and active site Lys as proposed in XometC [18].

PLP-dependent enzymes are very versatile enzymes, accounting for almost 4% of all classified enzyme activities. In the various classes of PLP-dependent enzymes, different amino acids located close to PLP were proposed as a catalytic base to deprotonate the amino group of incoming substrates. In XometC, ToCDS, and EcNifS_CsdB structures, the hydroxyl group of the PLP pyridine ring was more closely positioned to the substrate amino group than any other putative catalytic base amino acids. In all PLP-dependent enzymes, the transaldimination reaction should happen at least two times as coupled reactions of forward and backward at both sides of PLP and the forward and backward transaldimination reactions are symmetrical in the way that both amino groups of Lys side chain and substrate make the same Schiff-base linkage with PLP at the opposite sides. If we consider amino acid residues in the active site as a catalytic base as most enzymes do, two separate catalytic base residues are required at the both sides of PLP. However, the hydroxyl group of the PLP pyridine ring can perform the catalytic base role for the both sides at the center of two amino groups, which could be the simpler and more efficient model of PLP-dependent enzymes.

The GD structure allowed us to measure the dihedral angle in two opposite directions, towards the internal aldimine (a positive value) and the external aldimine (a negative value) (Table 2). In the IAA structure, the proposed dihedral angle with alanine was also measured. In the GD and IAA structures, the negative value of the external dihedral angles were almost conserved around −51°. In the active site of the ToCDS IAA structure, the alanine was tightly bound: the conserved Arg371 side chain had bifurcated hydrogen bonds with the carboxylate of alanine and Asn165 also had a hydrogen bond with the alanine carboxylate (Figure S4). The binding position of the incoming substrate cysteine would be similar to that of the product alanine in the IAA structure. The tightly bound fixed position of incoming amino group could maintain the dihedral angle in the external aldimine side of the GD and IAA structures. In the IAA structure, the three atoms of nitrogen in two amino groups and oxygen in the hydroxyl group of the PLP pyridine ring showed an almost perfect triangle shape. In the GD structure, the distance between the alanine amino group and the alanine-attached PLP carbon atom decreased as the substrate approached the PLP keeping the same external dihedral angle (Figure 6).

Crystal structure is the averaged structure of easily more than thousand billions of proteins arranged in crystal lattice. When substrate is provided, ToCDS enzymes in a crystal structure can exist as more than a catalytic state, for example as mixed catalytic states of two or more. We collected several tens of different ToCDS crystals for years by changing various variables like the duration of substrate incubation, the concentrations of substrate and ligands, and the ways of substrate soaking and cocrystallization. We speculate that the determination of catalytic intermediate structures of ToCDS was possible by capturing the moments when the enzymes of a certain catalytic state held the major population over the other states in the crystal.

Recent studies on the archaeal sulfur metabolism revealed many novel enzymes and pathways [14]. However, the information about the archaeal assimilatory sulfur metabolism for biomolecule synthesis such as [Fe-S] cluster assembly is limited. We do not understand the physiological role of ToCDS in *Thermococcus onnurineus* NA1. In the primary and tertiary structures, ToCDS has the highest similarity with EcSufS among the three compared EcCDSs. EcSufS belongs to the SUF system, which is involved in the [Fe-S] cluster synthesis under the adverse conditions of oxidative and heavy metal stress and iron starvation [34]. The environmental condition of *Thermococcus onnurineus* NA1's habitat is also physicochemically extreme with respect to temperature ranges of 2 to 100°C, oxygenation states, and fluid velocities [1]. In evolution, ToCDS might be the ancestor of EcSufS-like CDSs working under stress conditions. We need further studies to find out the physiological role of ToCDS.

## 5. Conclusions

We cloned the *ToCDS* gene of *Thermococcus onnurineus* NA1 and determined the crystal structures of ToCDS alone (NAT) and in complex with catalytic intermediate ligands (GD, IAA, and PSF). ToCDS belongs to class II CDS based on the primary and tertiary structures and showed the highest similarity with EcSufS. ToCDS is a PLP-dependent enzyme and the crystallographic snapshots of catalytic intermediates of ToCDS showed the conserved dihedral angle rotation of Schiff-base linkage relative to the PLP pyridine ring as shown in EcNifS_CsdB and XometC, which implies the PLP-dependent catalytic mechanism of ToCDS is well conserved. This study intends to help on understanding the catalytic mechanism of ToCDS and archaeal sulfur-trafficking system for the synthesis of sulfur-containing biomolecules.

## Supplementary Material

Figure S1. Omit maps of active site in ToCDS PSF, GD, and IAA structures.Figure S2. Active site in ToCDS GD and IAA structures.Figure S3. Proposed transaldimination mechanism from XometC structures.Figure S4. Tight recognition of alanine at the substrate-binding site of ToCDS.

## Figures and Tables

**Table 1:** Data collection and refinement statistics.

Data collection	NAT/NAT (crystal I)	GD/GD (crystal II)	IAA/GD (crystal III)	PSF/PSF (crystal IV)
PDB accession code	5B7S	5B87	5B89	5B7U
X-ray source	PAL7A	PAL5C	PAL5C	PAL5C
Space group	P2_1_2_1_2_1_	P2_1_2_1_2_1_	P2_1_2_1_2_1_	P2_1_2_1_2_1_
Unit-cell parameters a, b, c (Å)	67.0, 92.5, 145.4	67.1, 93.6, 145.0	62.9, 78.7, 171.9	62.8, 78.5, 171.3
Resolution range (Å)	50.0–2.6	50.0–2.3	50.0–1.5	50.0–1.9
Number of observed reflections (unique)	198,476 (28,968)	261,138 (40,955)	1,605,206 (128,579)	635,661 (66,011)
Completeness (%)	99.9 (100)	96.0 (100)	94.1 (91.9)	97.0 (91.9)
*R*_merge_ (%)	11.9 (41.6)	9.5 (38.0)	8.9 (56.6)	6.2 (30.6)
Average *I*/*σ* (*I*)	29.1 (7.1)	44.6 (5.3)	62.9 (6.5)	52.9 (12.2)
Refinement				
Resolution (Å)	36.4 (2.6)	49.4 (2.3)	49. 6 (1.5)	38.3 (1.9)
Number of reflection (*F*_obs_ > 0 *σ* (*F*_obs_))				
Working set	27,449	38,845	122,068	62,613
Free *R* set	1465	2040	6444	3326
*R*/*R*_free_ (%)^c^	19.3 (25.9)	17.7 (21.3)	16.7 (19.4)	16.5 (20.2)
Protein atom number	6270	6215	6256	6286
Ligand atom number				
Alanine	0	12	12	0
PLP	0	30	30	0
Cysteine	0	0	0	7
Isopropanol	0	0	0	4
Glycerol	0	0	6	0
1,2-Ethanediol	0	0	4	0
Water molecules	0	137	545	259
RMSD bond lengths (Å)	0.013	0.029	0.027	0.020
RMSD bond angles(^o^)	1.676	2.776	2.418	1.889
Mean B factors (Å^2^)				
Main-chain atoms	50.9	39.9	17.1	21.0
Side-chain atoms	56.2	46.4	22.3	26.6
Ligand				
Alanine	n.a.	51.8	32.1	n.a.
PLP	n.a.	32.5	22.9	n.a.
Cysteine	n.a.	n.a.	n.a.	46.6
Isopropanol	n.a.	n.a.	n.a.	36.7
Water atoms	n.a.	42.7	34.0	30.6
Glycerol	n.a.	n.a.	23.9	n.a.
1,2-Ethanediol	n.a.	n.a.	31.6	n.a.
Ramachandran plot (%)				
Favored regions	96.0	96.5	97.7	97.1
Allowed regions	2.9	2.8	1.8	2.4
Outlier regions	1.1	0.7	0.5	0.5

Values in parentheses are for the highest resolution shell. NAT represents the native structure, GD the gem-diamine structure, IAA the internal aldimine structure with a product alanine, and PSF the persulfide-bound structure. *R*_merge_ = ∑_*hkl*_∑_*i*_|(*I*_*i*_(*hkl*)) − 〈*I*(*hkl*)〉|/∑_*hkl*_∑_*i*_*I*_*i*_(*hkl*), where *I*_*i*_(*hkl*) is the mean intensity of *i*th observation of symmetry-related reflections *hkl*. *I*/*σ* (*I*) represents a signal to noise of reflections. *R*_free_ = ∑_*hkl*_||*F*_obs_| − |*F*_calc_||/∑_*hkl*_|*F*_obs_|, where *F*_calc_ is the calculated protein structure factor from the atomic model (*R*_free_ was calculated with randomly selected 5% of the reflections). *R* was calculated with the remaining 95% of the reflections in the same equation of *R*_free_. RMSD represents root-mean-square deviation. n.a.: not available.

**Table 2:** Dihedral angles of the PLP pyridine ring and Schiff-base linkage in ToCDS protomers.

Protomer	A (internal)	A (external)	B (internal)	B (external)
Crystal I (NAT/NAT)	47.7	n.a.	45.0	n.a.
Crystal II (GD/GD)	69.1	−50.2	64.8	−53.6
Crystal III (IAA/GD)	53.3	−51.4	64.9	−50.0
Crystal IV (PSF/PSF)	38.3	n.a.	50.3	n.a.

	Average	Standard dev.	Average	Standard dev.

NAT and PSF	45.3	5.2	n.a.	n.a.
GD	66.2	2.5	−51.2	2.0
IAA	53.3	n.a.	−51.4	n.a.

Positive dihedral angle values are for internal aldimine in the NAT, PSF, and GD structures and negative dihedral angle values are for external aldimine in the GD structures and proposed external aldimine in the IAA structure. n.a.: not available.

## Additional Points

*PDB References*. Atomic coordinates and structural factors for the reported crystal structures have been deposited in the Protein Data Bank under the accession codes of 5B7S (crystal I, NAT/NAT), 5B87 (crystal II, GD/GD), 5B89 (crystal III, IAA/GD), and 5B7U (crystal IV, PSF/PSF).
